# Transcriptional and Post-Transcriptional Mechanisms for Oncogenic Overexpression of *Ether À Go-Go* K^+^ Channel

**DOI:** 10.1371/journal.pone.0020362

**Published:** 2011-05-31

**Authors:** Huixian Lin, Zhe Li, Chang Chen, Xiaobin Luo, Jiening Xiao, Deli Dong, Yanjie Lu, Baofeng Yang, Zhiguo Wang

**Affiliations:** 1 Research Center, Montreal Heart Institute, Montreal, Quebec, Canada; 2 Department of Medicine, Universite de Montreal, Montreal, Quebec, Canada; 3 Department of Pharmacology (State-Province Key Laboratories of Biomedicine-Pharmaceutics of China), Harbin Medical University, Harbin, Heilongjiang, People's Republic of China; 4 Cardiovascular Research Institute (Key Laboratory of Cardiovascular Research, Ministry of Education of China), Harbin Medical University, Harbin, Heilongjiang, People's Republic of China; University of Houston, United States of America

## Abstract

The human *ether-à-go-go*-1 (h-eag1) K^+^ channel is expressed in a variety of cell lines derived from human malignant tumors and in clinical samples of several different cancers, but is otherwise absent in normal tissues. It was found to be necessary for cell cycle progression and tumorigenesis. Specific inhibition of h-*eag1* expression leads to inhibition of tumor cell proliferation. We report here that h-eag1 expression is controlled by the p53−*miR-34*−E2F1 pathway through a negative feed-forward mechanism. We first established E2F1 as a transactivator of h-*eag1* gene through characterizing its promoter region. We then revealed that *miR-34*, a known transcriptional target of p53, is an important negative regulator of h-eag1 through dual mechanisms by directly repressing h-eag1 at the post-transcriptional level and indirectly silencing h-*eag1* at the transcriptional level via repressing E2F1. There is a strong inverse relationship between the expression levels of *miR-34* and h-eag1 protein. H-*eag1*antisense antagonized the growth-stimulating effects and the upregulation of h-eag1 expression in SHSY5Y cells, induced by knockdown of *miR-34*, E2F1 overexpression, or inhibition of p53 activity. Therefore, p53 negatively regulates h-eag1 expression by a negative feed-forward mechanism through the p53−*miR-34*−E2F1 pathway. Inactivation of p53 activity, as is the case in many cancers, can thus cause oncogenic overexpression of h-eag1 by relieving the negative feed-forward regulation. These findings not only help us understand the molecular mechanisms for oncogenic overexpression of h-eag1 in tumorigenesis but also uncover the cell-cycle regulation through the p53−*miR-34*−E2F1−h-eag1 pathway. Moreover, these findings place h-eag1 in the p53−*miR-34*−E2F1−h-eag1 pathway with h-eag as a terminal effecter component and with *miR-34* (and E2F1) as a linker between p53 and h-eag1. Our study therefore fills the gap between p53 pathway and its cellular function mediated by h-eag1.

## Introduction

Abnormally enhanced proliferation often causes loss of control of cell growth leading to tumorigenesis or cancer formation. Several fundamental steps need to be fulfilled at the cellular level for tumorigenesis and these steps can be roughly viewed as characteristic alterations of some physicochemical processes: (1) cell volume, (2) intracellular Ca^2+^, and (3) intracellular pH. Evidence has emerged indicating a deregulated expression of ion channel protein-coding genes as well as ion channel malfunction as an important step in the development and progression of cancers. The ion channels critically related to cell proliferation and cancer are the K^+^ channels [1], [2].

Of various categories of K^+^ channels, the *ether à go-go* (*eag*) voltage-dependent K^+^ channel family stands out the most attractive one in relation to tumor generation, progression and metastasis [1]–[3]. *Eag1* (or Kv10.1 encoded by *KCNH1*), the founding member of the *eag* family, is restricted in its expression to the nervous system, indicating that the channel is not normally expressed in differentiated peripheral tissues. On the contrary, *eag1* is expressed in a variety of cell lines derived from human malignant tumors and in clinical samples of several different cancers [1]–[17], while the surrounding tissues are devoid of *eag1* expression. In these cell lines, *eag1* enhances the proliferation of the cells [13], and is required for the maintenance of growth. One of the most intriguing aspects of human *eag1* (h-*eag1*) channels is its relationship to cellular transformation; h-*eag1* channels are necessary for progression through the G_1_ phase and G_0_/G_1_ transition of the cell cycle [13]. Cells transfected with h-*eag1* are able to grow in the absence of serum, lose contact inhibition, and induce aggressive tumors when implanted into immune-depressed mice [13]. Moreover, specific inhibition of *eag1* expression by antisense technique [13], siRNA [18] or antibody [19] leads to a reduction in tumor cell proliferation *in vitro* and *in vivo*.

However, the molecular mechanisms underlying the oncogenic overexpression of h-eag1 remained unexplored. Expression of genes is tightly controlled by multiple factors at different levels. Transcription factors generally bind to the respective *cis*-acting elements, the protein-binding motifs, in the 5′-flanking region of a gene to switch on or off the transcription of the targeted gene [20]. In this way, transcription factors define the abundance of gene expression at the transcriptional level. Recently, microRNAs (miRNAs) have emerged as a new class of regulators of gene expression [21]. These small non-protein-coding mRNAs primarily elicit repression of protein translation by a partial complementary mechanism with its 5′end 2–8 nts, the “seed site”, base-paring the sequence motif(s) in the 3′ untranslated region (3′UTR) of target genes. In this way, miRNAs fine tune the expression of genes at the post-transcriptional level. These facts imply that transcription factors and miRNAs may somehow interplay and coordinate to control the expression of genes and better understanding of gene expression regulation should be addressed at both layers. This study was designed to decipher the molecular mechanisms underlying the oncogenic overexpression of h-eag1 by identifying the genomic structure and the key transcription factor that control the expression of h-*eag1* at the transcriptional level and by exploiting the potential role of miRNAs in fine-tuning the expression of h-eag1 at the post-transcriptional level.

## Results

**Figure 1:**
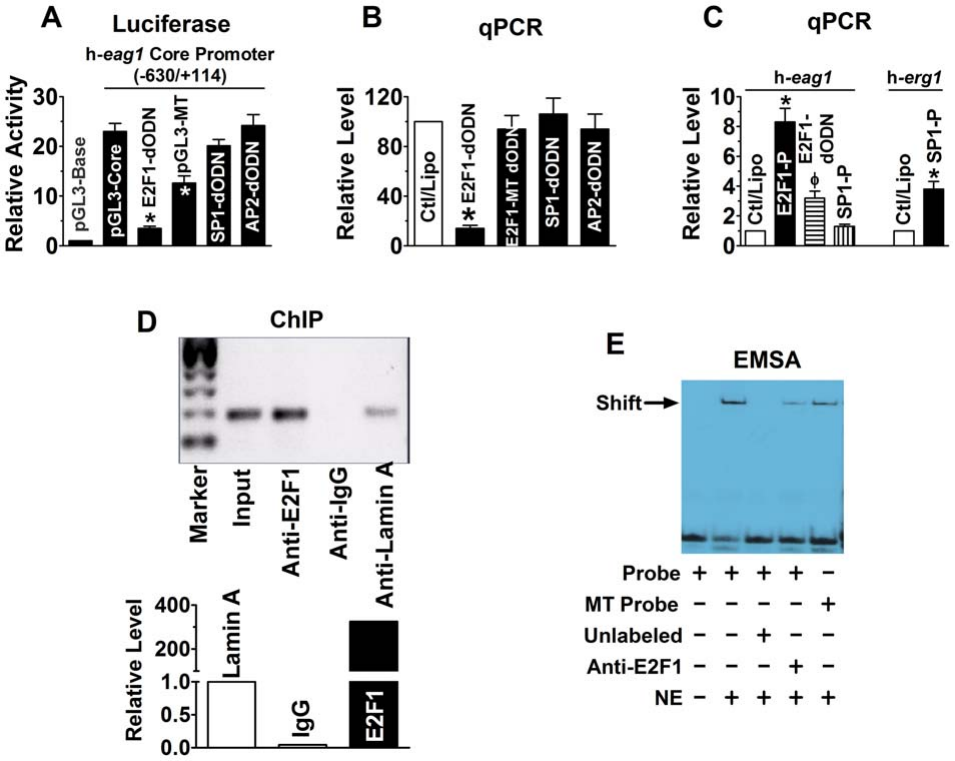
E2F1 as a transactivator of h-eag1 in SHSY5Y human neuroblastoma cells. (**A**) Role of E2F1 in driving the h-*eag1* core promoter activity. pGL3-Base: h-*eag1* promoter-free pGL3 vector for control; pGL3-Core: pGL3 vector carrying the h-*eag1* core promoter (a fragment spanning -630/+114); E2F1-dODN, SP1-dODN, and AP2-dODN: the decoy oligodeoxynucleotides targeting E2F1, SP1, and AP2 transcription factors, respectively, co-transfected with pGL3-Core; pGL3-Mutant: pGL3 vector carrying a mutated h-*eag1* core promoter. Transfection was carried out using lipofectamine 2000. *p<0.05 *vs* pGL3-Core; n = 5 for each group. (**B**) Changes of h-*eag1* mRNA level determined by real-time quantitative RT-PCR (qPCR) in SHSY5Y cells. E2F1-dODN, E2F1-MT dODN, SP1-dODN, or AP2-dODN was transfected alone. Ctl/Lipo: cells mock-treated with lipofectamine 2000; E2F1-MT dODN: the decoy oligodeoxynucleotides targeting E2F1 with mutation at the core region. *p<0.05 *vs* Ctl/Lipo; n = 5 for each group. (**C**) Increase in h-*eag1* mRNA level by overexpression of E2F1 in SHSY5Y cells transfected with the plasmid expressing the E2F1 gene. E2F1-P: pRcCMV-E2F1 expression vector (Invitrogen), the plasmid carrying the E2F1 cDNA. *p<0.05 *vs* Ctl/Lipo; n = 5 for each group. (**D**) Chromatin immunoprecipitation assay (ChIP) assay for the presence of E2F1 on its *cis*-acting elements in the h-*eag1* promoter region in SHSY5Y cells. Left panel: the bands of PCR products of the 5′-flanking region encompassing E2F1 binding sites following immunoprecipitation with the anti-E2F1 antibody or the anti-lamin A antibody for a negative control. Right panel: averaged data on the recovered DNA by anti-E2F1 expressed as fold changes over anti-lamin A band. Input: the input representing genomic DNA prior to immunoprecipitation. (**E**) Electrophoresis mobility shift assay (EMSA) for the fragment encompassing the putative E2F1 *cis*-acting element in the h-*eag1* promoter region to bind E2F1 protein in the nuclear extract from SHSY5Y cells. Probe: digoxigenin (DIG)-labeled oligonucleotides fragment containing E2F1 binding site; MT Probe: DIG-labeled fragment containing mutated E2F1 site at the core motif; NE: nuclear extract from SHSY5Y cells. Solid arrowhead points to the shifted band representing the DNA-protein complex. Note that the shifted band is weakened by anti-E2F1 antibody or with the mutant E2F1 binding motif.

In an initial effort to understand the molecular mechanisms for oncogenic overexpression of *eag1* in cancer cells, we characterized the promoter region of the gene. We used 5′RACE to identify the transcription start site (TSS) which was found located to 152 bp upstream the translation start codon (ATG) of h-*eag1* (GenBank accession No. DQ120124) (**Figure S1**). We then defined the minimal promoter region by luciferase reporter gene assay (**Figure S2**). Computer analysis revealed consensus sequences for E2F1, AP2, and SP1 within the core promoter region (position −630/+114), which might act as transactivators of h-*eag1* gene. Using the decoy oligodeoxynucleotide (dODN) approach [20], [22], [23], which contains the perfect binding site for the target transcription factor and can sequestrate the target leading to reduction of transcriptional activity (**Supporting Figures online**), we revealed a significant role of E2F1, but not of SP1 and AP2, in driving the core promoter activity (**Fig. 1A**). Mutation of the E2F1 *cis*-element rendered a loss of luciferase activity of the core promoter. We further verified E2F1 as a key factor in activating h-*eag1* transcription: E2F1-dODN decreased h-*eag1* mRNA level by ∼80% in SHSY5Y human neuroblastoma cells (**Fig. 1B**) and MCF-1 human breast cancer cells (**Figure S3**). With qPCR, we have also ruled out the role of SP1 and AP2 in transcriptional activation of h-*eag1* (**Fig. 1B**). Transfection of E2F1 plasmid, on the other hand, increased h-*eag1* mRNA level by as much as 8-fold, which was diminished by E2F1-dODN. As a negative control, transfection of SP1 plasmid did not significantly alter h-*eag1* mRNA level (**Fig. 1C**) despite that this maneuver was able to enhance expression of h-*erg1* at the mRNA level (**Fig. 1C**), another member of the *eag* K^+^ channel gene family, as already established in our previous study [24]. The ability of E2F1 to bind its *cis*-acting elements in the promoter region of h-*eag1* was verified using ChIP and EMSA (**Fig. 1D & 1E**). The transcription factor E2F1 plays a pivotal role in the coordinated expression of genes necessary for cell cycle progression and division, and is known to be an oncoprotein critical for the transcriptional activation of genes that control the rate of tumor cell proliferation [25]–[27]. Our finding thus indicates a role of E2F1 in oncogenic upregulation of h-*eag1* expression at the transcriptional level.

**Figure 2:**
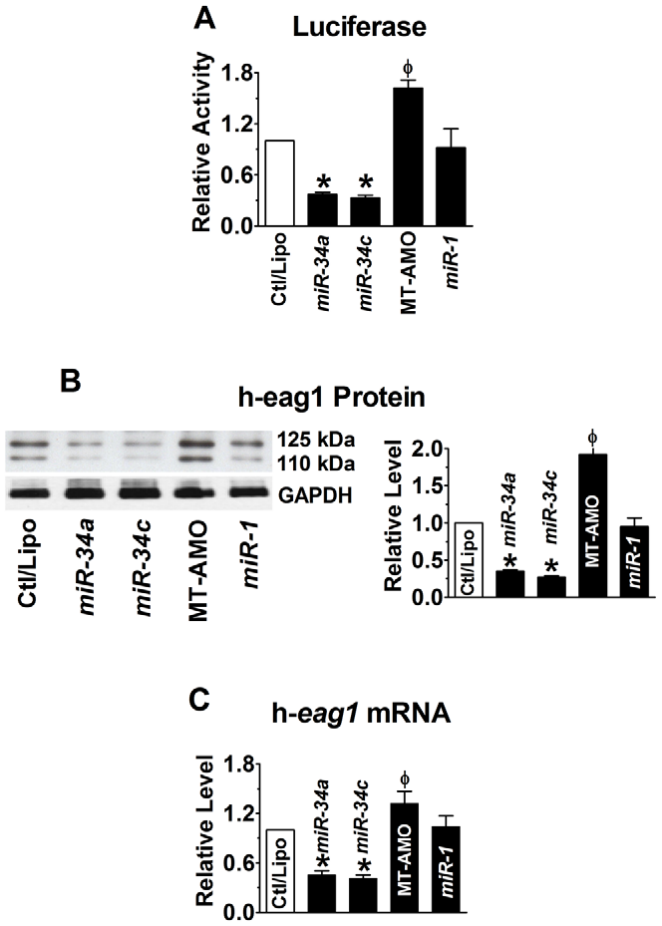
*miR-34* as a post-transcriptional repressor of h-eag1. (**A**) Repression of h-eag1 expression by *miR-34a* or *miR-34c*, as reported by luciferase activity assay with the pMIR-REPORT^TM^ luciferase miRNA expression reporter vector carrying the h-*eag1* 3′UTR in HEK293 cells. Ctl: cells transfected with the luciferase vector alone; MT-AMO: the multiple-target anti-miRNA antisense oligonucleotides to *miR-34a*, *miR-34b* and *miR-34c*, co-transfected with the luciferase vector and *miR-34a* or *miR-34c*. *p<0.05 *vs* Ctl/Lipo; ^φ^ *p*<0.05 *vs miR-34a*; n = 4 for each group. (**B**) Western blot analysis revealing repression of h-eag1 protein by *miR-34a* and *miR-34c* in SHSY5Y cells. *p<0.05 *vs* Ctl/Lipo; ^φ^ *p*<0.05 *vs miR-34a* alone; n = 4 for each group. The immunoblot bands shown were run on the same gel. (**C**) Effect of *miR-34* on h-*eag1* mRNA level in SHSY5Y cells. *p<0.05 *vs* Ctl/Lipo; ^φ^ *p*<0.05 *vs miR-34a* alone; n = 4 for each group.

We then investigated if h-*eag1* is also regulated at the post-transcriptional level by miRNAs. We first performed computational prediction of h-*eag1* as a target for miRNA regulation. And we identified multiple binding sites for a tumor-suppressor miRNA subfamily *miR-34* (including *miR-34a*, *miR-34b* and *miR-34c*) in the 3′UTR of h-*eag1* mRNA (**Figure S4**). To experimentally establish *miR-34*:h-*eag1* interaction, we inserted a fragment of 3′UTR of h-*eag1* containing the *miR-34* target sites into the position downstream the luciferase gene in the pMIR-REPORTTM vector. Transfection of *miR-34a* markedly suppressed the luciferase activities and the effect was reversed by their multiple-target anti-miRNA antisense oligonucleotides (MT-AMO) (**Fig. 2A** **; Supporting Figures online; Figure S5**), a single oligomer capable of targeting all three members of the *miR-34* subfamily [28]. Consistently, *miR-34a* decreased the protein level of h-eag1 by 70% in SHSY5Y cells, as assessed by Western blot analysis, whereas the MT-AMO increased it, presumably through downregulating the endogenous *miR-34a/b/c* (**Fig. 2B**). The h-eag1 antibody recognized two bands of 110 and 125 kDa, respectively, which according to the study by Napp et al [29] represent core-glycosylated form of eag1 protein and the complex *N*-Linked-glycosylated form of eag1, respectively. We analyzed the summation of the two bands to represent the total h-eag1 protein level and both bands were found affected by *miR-34* and MT-AMO. The same observations were expanded to *miR-34b* and *miR-34c* and to MCF-7 cells (**Figure S6 & S7**). Reduction of h-*eag1* expression was also seen at the mRNA level (**Fig. 2C**). Similar results were observed in MCF-7 cells (**Figure S7**). As a negative control, *miR-1* did not produce any appreciable effects on h-*eag1* expression.

**Figure 3:**
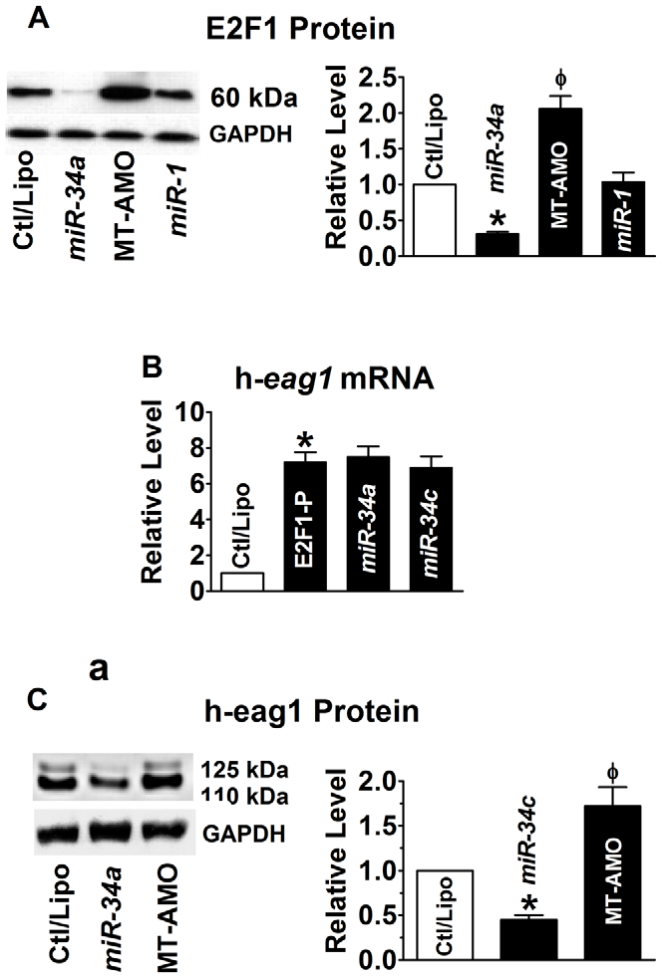
*miR-34* as a post-transcriptional repressor of E2F1. (**A**) Effect of *miR-34a* on E2F1 protein levels in SHSY5Y cells. *p<0.05 *vs* Ctl/Lipo; ^φ^ *p*<0.05 *vs miR-34a* alone; n = 6 for each group. (**B**) Inability of *miR-34* to affect the overexpression of h-*eag1* mRNA induced by transfection of the plasmid expressing the E2F1 cDNA. *p<0.05 *vs* Ctl/Lipo; n = 4 for each group. (**C**) Repression of h = eag1 protein levels by miR-34a in the presence of E1F1 overexpression by the vector containing the E2F1 cDNA. *p<0.05 *vs* Ctl/Lipo; ^φ^ *p*<0.05 *vs miR-34a* alone; n = 4 for each group.

It has been documented that *miR-34a* directly targets the mRNA encoding E2F1 and significantly reduces the levels of E2F1 and E2F3 proteins [30]–[32]. We confirmed that transfection of *miR-34a* reduced E2F1 protein levels by ∼68% in SHSY5Y cells (**Fig. 3A**) and the same results were obtained with *miR-34b* and *miR-34c* (**Figure S6 & S8**). Moreover, application of the MT-AMO caused significant increases in the protein levels of E2F1 (**Fig. 3A**) and h-eag1 (**Fig. 2B**). These results indicate that *miR-34* regulates h-*eag1* expression through at least two mechanisms. First, *miR-34* directly represses h-eag1 protein. Second, *miR-34* represses E2F1 protein, leading to reduced transcription of h-*eag1*. This latter effect also explains partially the effectiveness of *miR-34* to decrease h-*eag1* mRNA. This is supported by the experiments showing the lack of effects of *miR-34a* on h-*eag1* transcript level in cells co-transfected with the E2F1-carrying vector that does not contain the 3′UTR of E2F1 gene (**Fig. 3B**). By comparison, *miR-34a* retained its ability to repress h-eag1 protein in the presence of E2F1 overexpression (**Fig. 3C**).

**Figure 4:**
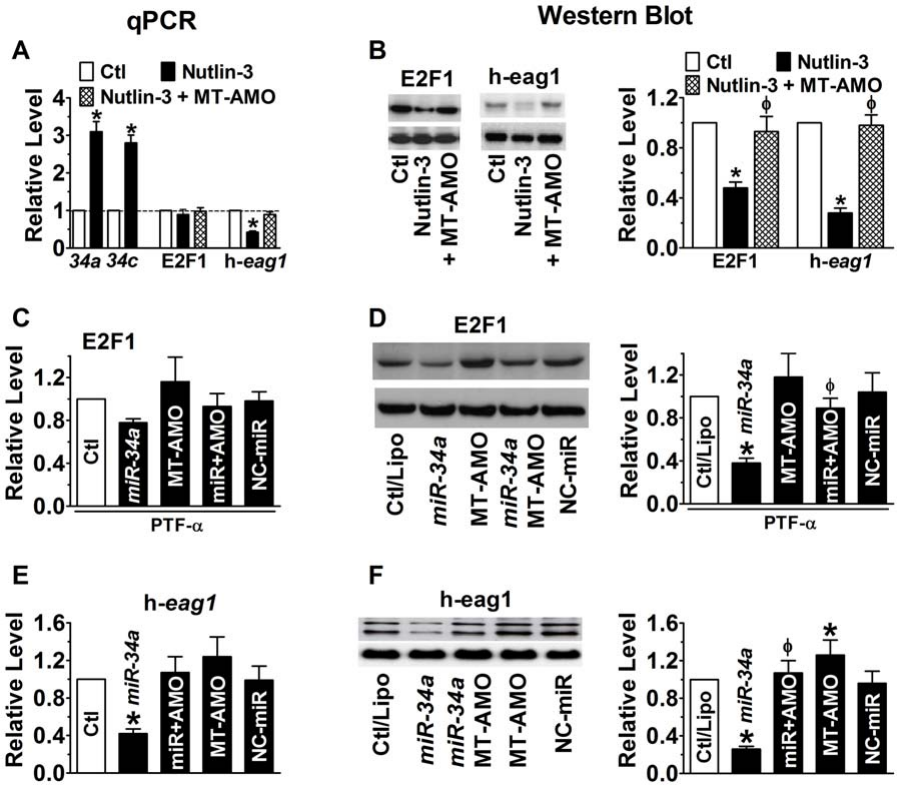
Anti-correlation between p53 activity and expression of E2F1 and h-eag1. (**A** & **B**) Effects of p53 activation by Mdm2 inhibitor nutlin-3 (1 µM) on expression of *miR-34*, E2F1 and h-*eag1* at mRNA and protein levels. SHSY5Y cells were pretreated with nutlin-3 and then transfected with MT-AMO. *p<0.05 *vs* Ctl/Lipo; ^φ^ *p*<0.05 *vs* Nutlin-3 alone; n = 4 for each group. (**C** & **D**) Downregulation of E2F1 at both mRNA and protein levels by *miR-34a* in the presence of p53 inhibitor Pifithrin-alpha (PFT-α; 30 µM). SHSY5Y cells were pretreated with PFT and then transfected with *miR-34a*. MT-AMO: an antisense oligomer to *miR-34a*, *miR-34b* and *miR-34c*; miR+AMO: co-transfection of *miR-34a* and MT-AMO; NC-miR: scrambled negative control miRNA. Control cells were mock-treated with lipofectamine 2000. *p<0.05 *vs* Ctl/Lipo; ^φ^ *p*<0.05 *vs miR-34a* alone; n = 4 for each group. (**E** & **F**) Downregulation of h-eag1 at both mRNA and protein levels by *miR-34a* in the presence of p53 inhibitor Pifithrin-alpha (PFT-α; 30 µM). The immunoblot bands shown were run on the same gel. *p<0.05 *vs* Ctl/Lipo; ^φ^ *p*<0.05 *vs miR-34a* alone; n = 4 for each group.

**Figure 5:**
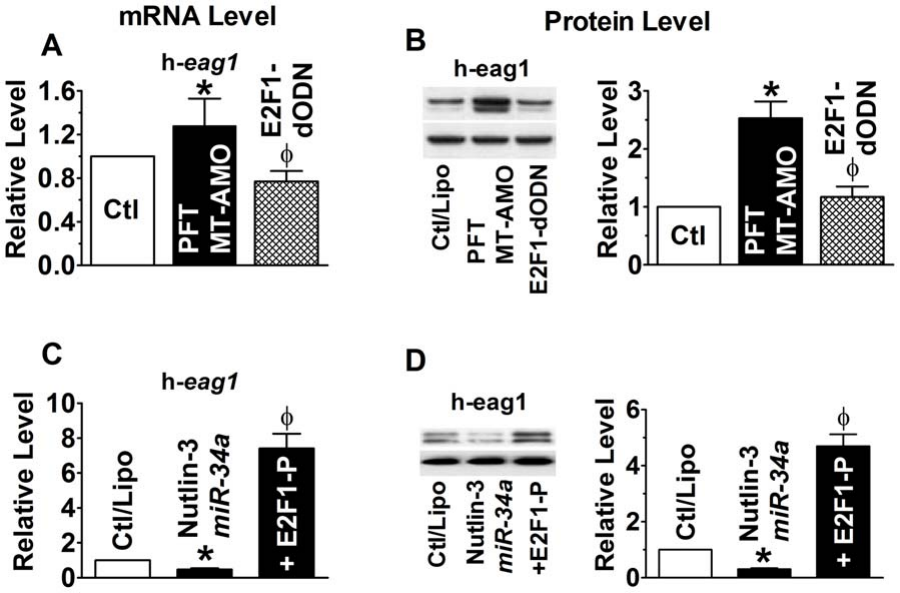
Control of h-*eag1* expression by E2F1. (**A** & **B**) Effect of E2F1 inhibition by its decoy oligodeoxynucleotides (E2F1-dODN) on h-*eag1* expression at both mRNA and protein levels in the presence of both p53 inhibitor and the MT-AMO to *miR-34*. SHSY5Y cells were pretreated with PFT (30 µM) and MT-AMO and then transfected with E2F1-dODN to sequestrate E2F1. *p<0.05 *vs* Ctl/Lipo; ^φ^ *p*<0.05 *vs* PFT+MT-AMO; n = 4 for each group. (**C** & **D**) Effect of E2F1 overexpression on h-*eag1* expression in the presence of both p53 activator and *miR-34a*. SHSY5Y cells were pretreated with nutlin-3 (1 µM) and *miR-34a* and then transfected with the plasmid to overexpress E2F1. *p<0.05 *vs* Ctl/Lipo; ^φ^ *p*<0.05 *vs* Nutlin-3+*miR-34a*; n = 4 for each group.

*miR-34* has been known to be a direct transcriptional target of p53 [33]–[36] and to mediate the apoptotic action of p53. Thus, changes of p53 activity are deemed to change the level of *miR-34* thereby those of E2F1 and h-eag1 as well. Indeed, p53 activation by Mdm2 inhibitor nutlin-3 (1 µM) increased *miR-34* level (**Fig. 4A**), and simultaneously decreased E2F1 and h-*eag1* mRNA concentrations (**Fig. 4A**) and protein levels (**Fig. 4B**). These changes were abrogated when the MT-AMO was co-applied with Nutlin-3. Pifithrin-alpha (PFT-α; 30 µM), the p53 inhibitor, produced exactly the opposite effects to p53 activator. Evidently, p53 negatively regulates expression of h-*eag1*. Moreover, in the presence of p53 inhibitor, exogenously applied *miR-34a* retained the full ability to downregulate E2F1 (**Fig. 4C & 4D**) and h-eag1 (**Fig. 4E & 4F**), suggesting that *miR-34* mediates the regulatory role of p53 on E2F1 and h-eag1. Furthermore, in the presence of both p53 inhibitor and the MT-AMO, h-*eag1* expression was markedly upregulated at both mRNA and protein levels, but the E2F1-dODN abolished these increases (**Fig. 5A & 5B**). On the other hand, the downregulation of h-*eag1* expression induced by co-application of p53 activator and *miR-34a* was abolished by E2F1 overexpression (**Fig. 5C & 5D**).

We reasoned that if E2F1 and *miR-34* are indeed important in the expression regulation of h-eag1, then we should see a positive correlation between E2F1 and eag1 levels and an inverse relationship between *miR-34* and h-*eag1* levels. Our data shown in **Figure S9** clearly demonstrate such relationships. It is noticed that in non-cancer cell lines HEK293 (human embryonic kidney cell), HMEC (human mammary epithelial cell) and HaVSMC (human aortic vascular smooth muscle cell), E2F1 levels are low and correspondingly, h-eag1 level is also low in these cells. In particular, in comparison with HMEC, several breast cancer cell lines (including 4T1 mouse mammary tumor cell line, and human breast cancer cell lines BT-20, MCF-7 and SkBr-3) express high levels of E2F1 and h-eag1 proteins.

**Figure 6:**
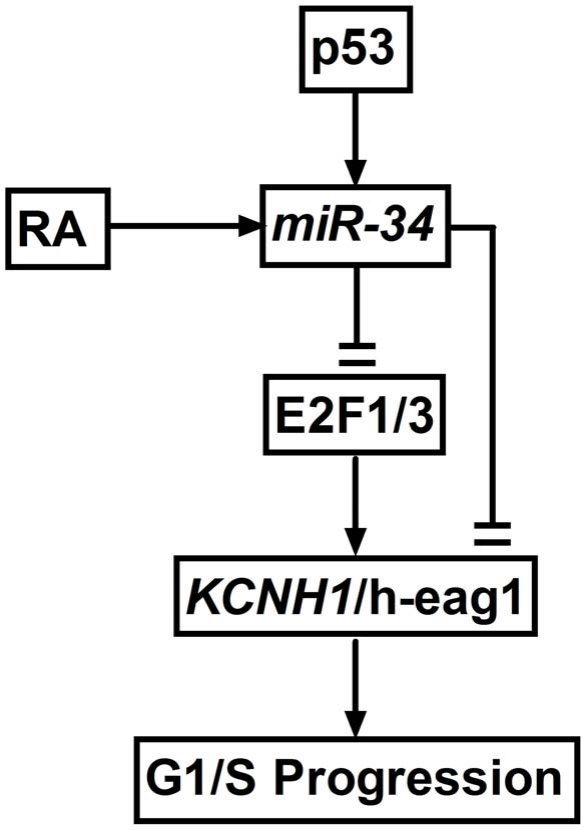
Proposed model of the p53−*miR-34*−E2F1−h-eag1 signaling pathway. RA: retinoic acid, which has been shown to enhance *miR-34* expression; E2F1/3: E2F1 and E2F3.

**Figure 7:**
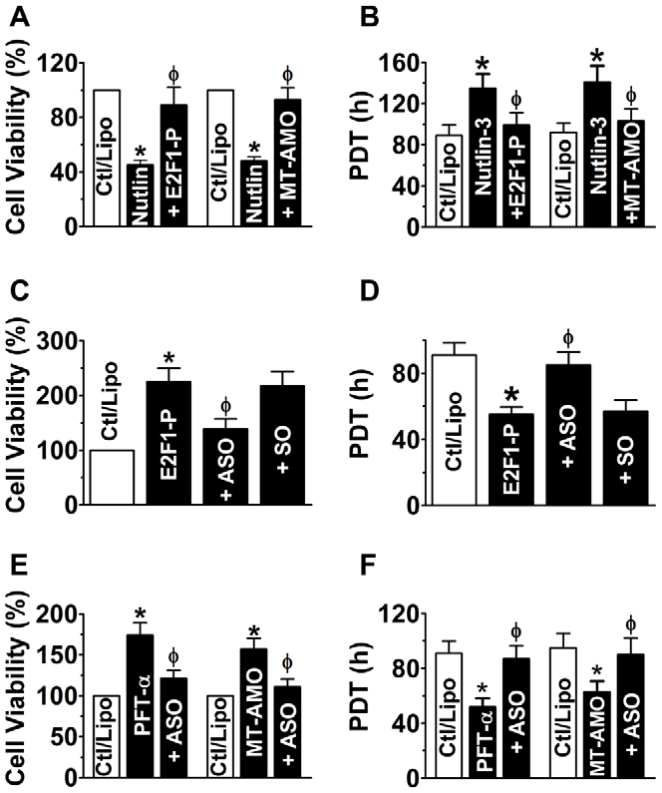
Effects of the p53−*miR-34*−E2F1−h-eag1 pathway on cell proliferation. (**A** & **B**) Effects of p53 activation by nutlin-3 (1 µM), E2F1 overexpression and *miR-34* knockdown on SHSY5Y cell proliferation evaluated with MTT assay (**A**) and by population doubling time (PDT) with flow cytometry methods (**B**). Cells were pretreated with nutlin-3 to activate p53 and then transfected with the plasmid carrying E2F1 cDNA for overexpression (E2F1-P) or MT-AMO to knockdown *miR-34*; control cells (Ctl/Lipo) were mock-treated with lipofectamine 2000. *p<0.05 *vs* Ctl/Lipo; ^φ^ *p*<0.05 *vs* Nutlin-3 alone; n = 4 for each group. (**C** & **D**) Effect of the antisense oligodeoxynucleotides (ASO) directed against h-*eag* gene on SHSY5Y cell growth induced by E2F1 overexpression, evaluated with MTT assay (**C**) and by PDT using flow cytometry methods (**D**). Cells were transfected with E2F1 plasmid alone (E2F1-P) or co-transfected with E2F1 plasmid and ASO (+ASO) or SO (sense oligomer for negative control; +SO). *p<0.05 *vs* Ctl/Lipo; ^φ^ *p*<0.05 *vs* E2F1-P alone; n = 4 for each group. (**E** & **F**) Effects of antisense to h-*eag1* (ASO) on cell-growth stimulation by PTF-α-induced p53 inactivation in SHSY5Y cells, determined by MTT (**E**) and by PDT (**F**). Cells were pretreated with PFT-α (30 µM) to inactivate p53 or transfected with MT-AMO, and then transfected with ASO; control cells (Ctl/Lipo) were mock-treated with lipofectamine 2000. *p<0.05 *vs* Ctl/Lipo; ^φ^ *p*<0.05 *vs* PFT-α alone; n = 5 for each group.

These above data allowed us to propose a new signaling pathway p53−*miR-34*−E2F1−h-eag1 (**Fig. 6**). Thus, we next sought to examine whether regulation of this pathway is related to the cell growth profile. We first demonstrated that activation of p53 by nutlin-3 induced a cell growth arrest in SHSY5Y cells, and overexpression of E2F1 alleviated the cell growth inhibition and so did transfection with the MT-AMO to knock down *miR-34* (**Fig. 7A & 7B**). On the other hand, the direct growth-stimulating effect of E2F1 was remarkably attenuated by inhibition of h-*eag1* with the antisense oligodeoxynucleotides directed against h-*eag1* gene but not by the sense oligomer for negative control (**Fig. 7C & 7D**). Further, inactivation of p53 by PFT-α or MT-AMO promoted cell growth, which was abrogated by the antisense to h-*eag1* (**Fig. 7E & 7F**).

## Discussion

The present study elucidated the molecular mechanisms underlying the oncogenic overexpression of h-eag1 at both transcriptional and post-transcriptional levels. The major finding includes identification of E2F1 as a key transcriptional activator of h-*eag1* and *miR-34* as an important translational inhibitor of h-eag1. In addition, we confirmed the targeting of E2F1 by *miR-34* and transactivation of *miR-34* by p53, and thus revealed the dual mechanisms by which *miR-34* controls expression of h-eag1: directly repressing h-eag1 at the post-transcriptional level and indirectly downregulating h-eag1 at the transcriptional level through repressing E2F1. Finally, we demonstrated that cell growth controls at the level of p53, *miR-34* or E2F1 were related to h-eag1 expression.

Based on our findings, we were able to establish a novel signaling pathway: p53−*miR-34*−E2F1− h-eag1. It appears that h-eag1 is a terminal effecter component in the p53−*miR-34*−E2F1 pathway for expression regulation and functional signaling. When p53 activity increases in response to environmental and cellular stresses, *miR-34* is deemed to increase, and the increased *miR-34* will decrease E2F1 to diminish h-*eag1* gene transcription and will also repress h-eag1 protein translation as well; diminishment of h-eag1 expression and function then results in a shut-down of cell proliferation or a cell cycle arrest. This implies that h-eag1 executes the cell-cycle checkpoint signal from p53 transmitting along the p53−*miR-34*−E2F1−h-eag1 pathway (**Fig. 6**). Indeed, expression of h-*eag1* is cell cycle-related; upon synchronization of the cells in G_1_ phase with retinoic acid, eag1 current amplitude decreased to less than 5% of the control [13]. And retinoic acid has been showed to stimulate expression of *miR-34a* accompanied by a decrease in E2F1 protein level [32]. These findings may be extended to h-erg-1 K^+^ channel (or HERG): h-erg1 may also be a component of the p53−*miR-34*−E2F1 pathway, according to our data shown in Figures S3, S8 and S9.

The tumor-suppressor gene p53 and its downstream genes consist of a complex molecular signaling network and p53 is at the center of this network regulating diverse physiological responses to cancer-related stresses. Activated p53 in response to DNA damage or oncogene activation induces cell cycle arrest, which can be transient or permanent (senescence), or promotes apoptosis in cases where the damage is too severe; conversely, inactivation of p53 causes oncogenic cell growth. Our study herein revealed that *miR-34*, a known transcriptional target of p53, is an important negative regulator of h-eag1 through dual mechanisms by direct repression at the post-transcriptional level and indirect silencing at the transcriptional level via post-transcriptionally repressing E2F1 that we have established to be a transactivator of h-*eag1*. p53 activates *miR-34* transcription; upregulation of *miR-34* represses E2F1 and h-eag1; repression of E2F1 downregulates expression of h-*eag1*. Therefore, p53 negatively regulates h-eag1 expression by a negative feed-forward mechanism through the p53−*miR-34*−E2F1 pathway and inactivation of p53 activity as it is the case in many cancers can thus cause oncogenic overexpression of h-eag1 by relieving the negative feed-forward regulation. These findings not only help us understand the molecular mechanisms for oncogenic overexpression of h-eag1 in tumorigenesis but also uncover the cell-cycle regulation through the p53−*miR-34*−E2F1−h-eag1 pathway. Moreover, these findings place h-eag1 in the p53−*miR-34*−E2F1−h-eag1 pathway with h-eag as a terminal effecter component and with *miR-34* (and E2F1) as a linker between p53 and h-eag1. Our study therefore fills a gap between p53 pathway and its cellular function mediated by h-eag1.

Intriguingly, it has been demonstrated that in vertebrates *miR-34* is initially expressed widespread throughout the brain in early stage of development and expression becomes limited to the anterior region of the hindbrain in later stages [37]. In adult, *miR-34* is absent from forebrain and midbrain and present only in the caudal ventral and lateral isthmus and hindbrain nuclei [38]. This low expression of *miR-34* may partially underlie the high abundance of eag1 in brain [39]. Moreover, this low level of *miR-34* may also explain the enriched expression of E2F1 in brain [40]. Further, analysis of human specimens showed that *miR-34a* expression is down-regulated in glioblastoma tissues as compared with normal brain [41]. This can well result in upregulation of h-eag1 during in tumorigenesis of brain. Indeed, downregulation of *miR-34* has been found in a wide spectrum of tumors [36] in one hand and upregulation of h-eag1 in cancer tissues on the other hand, consistent with h-eag1 being a CNS-localized voltage-gated K^+^ channel that is ectopically expressed in a majority of extracranial solid tumors [39]. Recent studies have shown that members of the *miR-34* family possess anti-proliferative potential and induce cell cycle arrest, senescence, and/or apoptosis [31]–[36]. Downregulation of miR-34 should produce the opposite changes and upregulation of h-eag1 may mediate the cell growth-promoting effect of miR-34 downregulation.

## Materials and Methods

### Rapid amplification of cDNA ends (5′RACE)

The transcription start site (TSS) of h-*eag1* gene was determined with Ambion′s FisrtchoiceTM RNA–Ready cDNA Human Brain RNA ligase-mediated 5′RACE kit, as previously described [24], [42], [43]. Human brain RNA sample was purchased from Clontech. The gene specific primers (GSP) were designed based on the human *KCNH1* cDNA (GenBank accession HSU04270) for h-*eag1*; GSP1 5′-TTCACGGGCACCACATCCAC-3′ (corresponding to 511–530 bp) and GSP2 (nest primer): 5′-CCGAGCGTTGGCGATGATGA-3′ (corresponding to 268–298 bp).

### PCR amplification of putative promoter regions and construction of promoter-luciferase fusion plasmids

A series of 3′ to 5′deletions were amplified with human genomic DNA (Homo sapiens BAC clone RP11-543B16) as a template. PCR products were subcloned into luciferase-containing pGL3-Basic (Promega) vector [24], [42], [43]. *Cis*-acting elements for transcription factor (TF) binding sites were analyzed with *MatInspector* V2.2.

### Decoy oligodeoxynucleotides for E2F1 (E2F1-dODN)

Single-stranded phosphorothioate oligodeoxynucleotides were synthesized by Integrated DNA Technologies, Inc. (Coralville, IA). The ODNs were washed in 70% ethanol, dried, and dissolved in sterilized Tris-EDTA buffer (10 mM Tris and 1 mM EDTA). The supernatant was purified using Micro Bio-Spin 30 columns (Bio-Rad Laboratories, Hercules, CA) and quantified by spectrophotometry. The double-stranded E2F1-dODN was then prepared by annealing complementary single-stranded oligodeoxynucleotides by heating to 95°C for 10 min followed by cooling to room temperature (RT) slowly over 2 h [20], [24], [42]–[44]. The E2F1-dODN sequences are:

5′-GCCCTC**TTCGCGCC**TCCCTCC-3′

3′-CGGGAGAAGCGCGGAGGGAGG-5′; the mutated E2F1-dODN sequences are:

5′-GCCCTC**TTCGatCC**TCCCTCC-3′

3′-CGGGAGAAGCtaGGAGGGAGG-5′ (the boldface and underlined letters indicate the core binding motif for E2F1 and the mutated nucleotides are in lower case).

### Antisense oligodeoxynucleotides to h-*eag*1 gene

Following the study reported by Pardo *et al* [14], a 19mer antisense phosphorothioate oligodeoxynucleotides (ASO, 5′-CAGCCATGGTCATCCTCCC-3′) spanning the putative initiation codon of h-*eag1* was used to test proliferation inhibition. The sense oligodeoxynucleotides (SO, 5′-GGGAGGATGACCATGGCTG-3′) was used as a negative control.

### Synthesis of miRNAs and anti-miRNA antisense inhibitors

*miR-34a, miR-34b,* and *miR-34c* (**Figure S4**), and their antisense inhibitor oligonucleotides (MT-AMO) (**Figure S5**) were synthesized by Integrated DNA Technologies, Inc. (IDT). The MT-AMO tested in this study was designed to integrate the AMOs against *miR-34a*, *miR-34b* and *miR-34c* into one AMO unit. An eight-nucleotide linker was inserted to connect the two adjacent antisense units and five nucleotides at both ends were locked with methylene bridges (LNA), with the rest of residues at the form of DNA.

### Construction of chimeric miRNA target site−luciferase reporter gene vectors

To construct reporter vectors bearing miRNA-target sites, the 3′UTRs of h-*eag1* and h-*erg1* genes were inserted into the cloning site downstream the luciferase gene in the pMIR-REPORTTM luciferase miRNA expression reporter vector (Ambion, Inc) [24], [42]–[45].

### Mutagenesis

Base-substitution mutations were made to the E2F1 binding site in the promoter region the h-*eag1* gene by PCR-based methods [20]. The wild-type sequence is:

••••••TACCCTCGCGCCCTCTTCGCGCCTCCCTCCCTGCGGCCCG••••••

••••••ATGGGAGCGCGGGAGAAGCGCGGAGGGAGGGACGCCGGGC••••••, and the mutant sequence is (mutated nucleotides are shown by the underlined lower-case letters):

••••••TACCCTCGCGCCCTC**ga**CG**at**CCTCCCTCCCTGCGGCCCG••••••

••••••ATGGGAGCGCGGGAGctGCtaGGAGGGAGGGACGCCGGGC••••••. These sequences were cloned into the pGL3 vector for luciferase activity assays.

### Cell culture

The cell lines used in this study were all purchased from American Type Culture Collection (ATCC, Manassas, VA). SHSY5Y human dopaminergic neuroblastoma cells with wild-type p53 status, MCF-7 human breast cancer cells with wild-type p53 status and HEK293 human embryonic kidney cells were cultured in Dulbecco's Modified Eagle Medium (DMEM). The cultures were supplemented with 10% fetal bovine serum and 100 µg/ml penicillin/streptomycin (Invitrogen).

### Transfection and Luciferase Assay

For promoter activity measurements, HEK293 cells (1×10^5^/well) were transfected with 1 µg pGL3–target DNA (firefly luciferase vector) and 0.1 µg PRL-TK (TK-driven Renilla luciferase expression vector), along with decoy ODNs or other constructs, using lipofectamine 2000 (Invitrogen, Carlslbad, CA). For miRNA experiments, HEK293 cells (1×10^5^/well) were transfected with pMIR-REPORTTM vector along with miRNA mimics, antisense ODNs, alone or together as to be specified. For E2F1 overexpression, pRcCMV-E2F1 expression vector (Invitrogen) along with other constructs as specified was transfected. The transfection procedures took place 24 h after starvation of cells in serum-free medium. Following transfection (48 h), luciferase activities were measured with a dual luciferase reporter assay kit (Promega) on a luminometer (Lumat LB9507), as described previously [42]–[47].

### Western blot analysis

Following 48 h treatments, protein samples were extracted by following the procedures essentially the same as described in detail elsewhere [42]–[47]. The protein content was determined with Bio-Rad Protein Assay Kit (Bio-Rad, Mississauga, ON, Canada) using bovine serum albumin as the standard. Protein sample (∼30 µg) was fractionated by SDS-PAGE (7.5%–10% polyacrylamide gels) and transferred to PVDF membrane (Millipore, Bedford, MA). The sample was incubated overnight at 4°C with the polyclonal anti-rabbit primary antibodies for E2F1 (Santa Cruz) and h-eag1 (Alomone Labs), diluted 1:1000 in TBS containing 3% BSA. Bound antibodies were detected using the chemiluminescent substrate (Western Blot Chemiluminescence Reagent Plus, NEN Life Science Products, Boston, USA). Western blot bands were quantified using QuantityOne software by measuring the band intensity (Area × OD) for each group and normalizing to GAPDH (anti-GAPDH antibody from Research Diagnostics Inc) as an internal control. The final results are expressed as fold changes by normalizing the data to the control values.

### Real-time RT-PCR quantification of mRNA and miRNA levels

For quantification of transcripts of E2F1 and h-*eag1*, conventional real-time RT-PCR was carried out with total RNA samples extracted from SHSY5Y human neuroblastoma cells 24 h after treatments and treated with DNase I. TaqMan quantitative assay of transcripts was performed with real-time two-step reverse transcription PCR (GeneAmp 5700, PE Biosystems), involving an initial reverse transcription with random primers and subsequent PCR amplification of the targets. Expression level of GAPDH was used as an internal control.

The *mir*Vana™ qRT-PCR miRNA Detection Kit (Ambion) was used in conjunction with TaqMan real-time PCR for quantification of miRNA transcripts in our study, following the manufacturer's instructions. The total RNA samples were isolated with Ambion's *mir*Vana miRNA Isolation Kit. Reactions contained *mir*Vana qRT-PCR Primer Sets specific for human *miR-34a*, *miR-34b, miR-34c*, *miR-1* (as a negative control) and human 5S rRNA (as a positive control). qRT-PCR was performed on a 96-well StepOnePlusTM system (A&B Applied Biosystems) for 40 cycles. We first determined the appropriate cycle threshold (Ct) using the automatic baseline determination feature. We then performed dissociation analysis (melt-curve) on the reactions to identify the characteristic peak associated with primer-dimers in order to separate from the single prominent peak representing the successful PCR amplification of miRNAs. Fold variations in expression of miRNAs between RNA samples were calculated [42]-[47].

### Chromatin immunoprecipitation assay (ChIP)

ChIP assays were conducted with the EZ ChIP kit according to the manufacturer's instructions (Upstate Cell Signaling Solutions, Lake Placid, NY) [24]. Briefly, SHSY5Y cells were grown to subconfluency, washed and fixed in 1% formaldehyde for 10 min to crosslink nucleoprotein complexes and scraped in phosphate buffered saline containing protease inhibitor cocktail. Pelleted cells were then lysed and sonicated in detergent lysis buffer. Sheared DNA–protein complexes were immunoprecipitated by incubating overnight the lysates with 2 µg antibodies against E2F1 or lamin A (as a control). Protein A/G Plus beads (Santa Cruz) were used, and after extensive washing, crosslinks were removed at 65°C over overnight in an elution buffer (1% SDS, 0.1 M NaHCO_3_). The DNA was isolated using the QIAquick PCR purification kit (Qiagen) and the presence of the h-*eag1* promoter was analyzed by PCR amplification using 10% of purified DNA. The primers used for h-*eag1* promoter sequence containing E2F1 *cis*-elements were 5′GAGACCCTCACTCAGACGCA3′ (forward) and 5′CAGCACTAGGCTTCGGGTGG3′ (reverse). The PCR products were analyzed by gel electrophoreses on an 8% non-denaturing polyacrylimide gel and subsequent ethidium bromide staining.

### Electrophoretic mobility shift assay (EMSA)

EMSA was performed with the DIG Gel Shift kit (Roche, Mannheim, Germany). Varying amounts of nuclear protein extracts from SHSY5Y cells were incubated with digoxigenin (DIG)-labeled double-stranded oligonucleotides containing the putative E2F1 *cis*-acting elements. For competition experiments, 100-fold excess of unlabeled double-stranded E2F1 consensus oligonucleotides, and for super-shift experiments, 1 µg of E2F1 antibody (Santa Cruz Biotechnology, Inc., Santa Cruz, CA), were added to the reaction. The generated chemiluminescent signals were recorded on the X-ray film [20], [24].

### Drug treatment

For drug treatment, cells were starved in serum-free medium for 12 h and then incubated with the drugs at desired final concentration for 1 h, followed by transfection of various constructs. The drugs used included doxorubicin hydrochloride (p53 activator, Biovision Research Products, Mountain View, CA) and Pifithrin-alpha (PFT-α, p53 inhibitor; Cedarlane Lab Ltd, Homby, ON). For experiments involving co-application of activators and inhibitors, cells were pretreated with inhibitors for 5 h before addition of activators.

### MTT assay for cell proliferation

The WST-1 kit (Roche, Penzberg, Germany). In brief, 24 h after treatment with varying constructs, cells were washed with PBS and grown in 100 µl of fresh culture medium plus 10 µl of WST-1 reagent for 30 min. The absorbance was measured at 425 nm using a Spectra Rainbow microplate reader (Tecan, Grödig, Austria) with a reference wavelength of 690 nm [20], [47], [48].

### Determination of population doubling time (PDT)

Cell proliferation was assessed by characterizing the log phase growth with population doubling time (PDT) calculated by using the equation: 1/(3.32 × (log*N*_H_ - log*N*_I_)/(*t*_2_ - *t*_1_), where *N*_H_ is the number of cells harvested at the end of the growth period (*t*_2_, 24 h) and *N*_I_ is the number of cells at 5 h (*t*_1_) after seeding [48].

### Statistical analysis

Group data are expressed as mean ± S.E. Statistical comparisons (performed using ANOVA followed by Dunnett's method) were carried out using Microsoft Excel. A two-tailed *p*<0.05 was taken to indicate a statistically significant difference. *Cis*-elements for transcription factor binding sites were analyzed with *MatInspector* V2.2.

## Supporting Information

Figure S1 5′-flanking region containing the core promoter sequence of the h-*eag1* (KCNH1) gene. The transcription start site (TSS) is indicated by a backward arrow and designated position -1. The TATA box and the consensus binding sequences for SP1, AP2, and E2F transcription factors are underlined and the core sequences of the *cis*-acting elements are bold. For convenience, the E2F consensus sites are numbered in order from TSS and the positions, relative to TSS, at the first nucleotide of the consensus core sequence are donated by the numbers in the brackets.
(PDF)

Figure S2 Analysis of the h*-eag1* promoter activity in various cell lines. A schematic representation of the 5′ deletion constructs of the h*-eag* promoter region is shown on the left panel. Nucleotides of fusion plasmids are numbered with respect to the TSS (-1) identified by 5′RACE. Firefly luciferase expression levels were divided by co-expressed Renilla luciferase activity and expressed as relative activity divided by the promoter-less construct (pGL3-Basic). Shown is comparison of the h*-eag1* promoter activities expressed in three human cancer cell lines neuroblastoma SHSY5Y, breast cancer MCF-7 and embryonic kidney cell HEK293, and mouse atrial tumor cell line HL-1. The data were averaged from 5 experiments in duplicate for each cell.
(PDF)

Figure S3 E2F1 as a transactivator of h-eag1 in MCF-7 human breast cancer cell line. (**A**) Role of E2F1 in driving the h-*eag1* core promoter activity. pGL3-Base: h-*eag1* promoter-free pGL3 vector for control; pGL3-Core: pGL3 vector carrying the h-*eag1* core promoter (a fragment spanning -630/+114); E2F1-dODN, SP1-dODN, and AP2-dODN: the decoy oligodeoxynucleotides targeting E2F1, SP1, and AP2 transcription factors, respectively, co-transfected with pGL3-Core; pGL3-Mutant: pGL3 vector carrying a mutated h-*eag1* core promoter. Transfection was carried out using lipofectamine 2000. *p<0.05 *vs* pGL3-Core; n = 4 for each group. (**B**) Changes of h-*eag1* mRNA level determined by real-time quantitative RT-PCR (qPCR). E2F1-dODN, E2F1-MT dODN, SP1-dODN, or AP2-dODN was transfected alone. Ctl/Lipo: cells mock-treated with lipofectamine 2000; E2F1-MT dODN: the decoy oligodeoxynucleotides targeting E2F1 with mutation at the core region. *p<0.05 *vs* Ctl/Lipo; n = 4 for each group. (**C**) Increase in h-*eag1* mRNA level by overexpression of E2F1 in MCF-7 cells transfected with the plasmid expressing the E2F1 gene. E2F1-P: pRcCMV-E2F1 expression vector (Invitrogen), the plasmid carrying the E2F1 cDNA. *p<0.05 *vs* Ctl/Lipo; n = 4 for each group.
(PDF)

Figure S4 Multiple complementary motifs between each of the three isoforms of has-*miR-34* and the 3′UTRs of h*-eag1* mRNA (A) and h-*erg1* mRNA (**B**). Matched nucleotides are in boldface and linked by “|”, and wobble matches are indicated by “:”.
(PDF)

Figure S5 The multiple-target anti-miRNA antisense oligonucleotide fragment (MT-AMO) used to knock down all three different isoforms of has-*miR-34* (*miR-34a, miR-34b* and *miR-34c*).
(PDF)

Figure S6 Effects of *miR-34b* and *miR-34c* on expression of E2F1 (**A**) and h-eag1 (**B**) at the protein level in SHSY5Y cells, assessed by Western blot analysis. Control cells were mock-treated with lipofectamine 2000. *miR-1* was used as a negative control. MT-AMO: an antisense oligomer to *miR-34a*, *miR-34b* and *miR-34c*; +MT-AMO: co-app;lication of *miR-34c* and MT-AMO. *p<0.05 *vs* Ctl/Lipo; ^ϕ^ *p*<0.05 *vs miR-34c* alone; n = 4 for each group.
(PDF)

Figure S7 *miR-34* as a post-transcriptional repressor of h-eag1 in MCF-7 human breast cancer cells. (**A**) Western blot analysis revealing repression of h-eag1 protein by *miR-34a*. *p<0.05 *vs* Ctl/Lipo; ^ϕ^ *p*<0.05 *vs miR-34a* alone; n = 5 for each group. (**B**) Effect of *miR-34a* on h-*eag1* mRNA level. *p<0.05 *vs* Ctl/Lipo; ^ϕ^ *p*<0.05 *vs miR-34a* alone; n = 5 for each group.
(PDF)

Figure S8 Multiple complementary motifs between each of the three isoforms of has-*miR-34* and the 3′UTR of E2F1 mRNA. Matched nucleotides are in boldface and linked by “|”, and wobble matches are indicated by “:”.
(PDF)

Figure S9 Expression correlations between E2F1 and h-eag1 and between *miR-34* and h-eag1. (**A**) Western blot analysis showing a positive correlation between E2F1 protein and h-eag1 protein levels in various cancer and non-cancer cell lines as specified. Fill circles are experimental data, straight line represents linear regression and dashed lines define the 95% confidence range. The correlation coefficient (r^2^) and the slope are indicated. (**B**) qPCR analysis showing an inverse relationship between *miR-34a/b* and h-eag1 protein levels in HMEB, HaVSMC, SkBr-3, SHSY5Y, U2OS, HT29, and Sk-28 cells. Fill circles are experimental data, straight line represents linear regression and dashed lines define the 95% confidence range. The correlation coefficient (r^2^) and the slope are indicated.
(PDF)
