# Correction: Transcriptional and Post-Transcriptional Mechanisms for Oncogenic Overexpression of *Ether À Go-Go* K^+^ Channel

**DOI:** 10.1371/annotation/45b3e6bc-1065-4357-b215-465176dcc269

**Published:** 2011-11-23

**Authors:** Huixian Lin, Zhe Li, Chang Chen, Xiaobin Luo, Jiening Xiao, Deli Dong, Yanjie Lu, Baofeng Yang, Zhiguo Wang

It has been brought to the attention of the PLoS ONE Editors that Figure 1d in this article is a duplication of Figure 3a included in the authors’ previous publication: Xiao J, Lin H, Luo X, Luo X, Wang Z miR-605 joins p53 network to form a p53:miR-605:Mdm2 positive feedback loop in response to stress..EMBO J. 2011 Feb 2;30(3):524-32. Epub 2011 Jan 7.

The authors would like to apologize to readers and to the editors of EMBO Journal for this error. The corrected Figure 1d that should have been included in the PLoS ONE article can be viewed here: 

**Figure:**
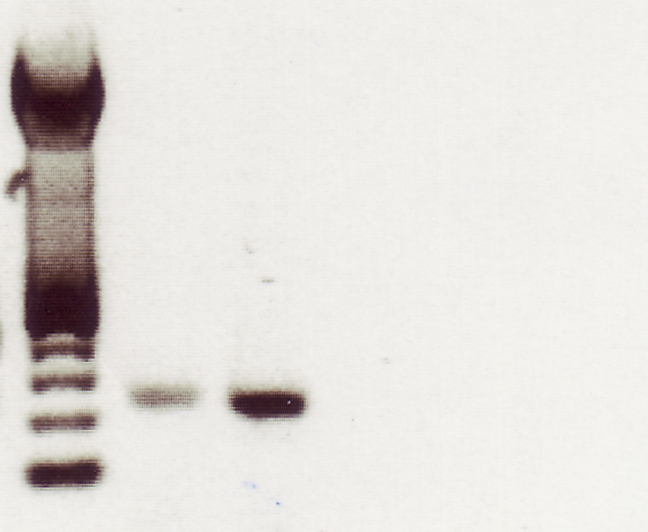


The PLoS ONE Editors have followed up with the Montreal Heart Institute Research Centre which, after an investigation, indicated that the duplication of the figure was caused by a mistake during the preparation of the manuscript. 

