# Supplementary material for: Transcriptional and Post-Transcriptional Mechanisms for Oncogenic Overexpression of Ether À Go-Go K+ Channel
Source: PLoS One. 2011 May 31;6(5):e20362. doi: 10.1371/journal.pone.0020362 (PMC3105031; doi:10.1371/journal.pone.0020362)
Supplement: Figure S1 — 5′-flanking region containing the core promoter sequence of the h-eag1 (KCNH1) gene. The transcription start site (TSS) is indicated by a backward arrow and designated position -1. The TATA box and the consensus binding sequences for SP1, AP2, and E2F transcription factors are underlined and the core sequences of the cis-acting elements are bold. For convenience, the E2F consensus sites are numbered in order from TSS and the positions, relative to TSS, at the first nucleotide of the consensus core sequence are donated by the numbers in the brackets. (PDF) [file pone.0020362.s001.pdf]

Supplementary Figures

Figure S1

1 GAGAGATTTC TGGGCAAGAA TTAGTTCTTC TGTTTTTGAA ATTAATTAAC TGAGACAGGA

....

541 CATTTGTGTC TACGGTAAAA ACAACCCAAG ACAACTCTTC TGAAGCTGAA TCTGCCAGTC

601 ACTC**TAAAT**C TGCACTATTG CAATACGACT TCATTAATTA CTGCTCAAAC ACTGGCCTAG

TATA Box

....

901 GTGGCGCTGC CGTCCCAGCC TTGGTCCACG GCCGCCCGCC ACTTCCCTGC CTCTGCGGGA

961 GCCCCCCAGC TTGGTCTCCC GCAGGCCGGC GGTGGGGCAC GAGCCCGGTA GGGGGCGCCG

1021 CCGGCCCAGG CGCCGCTGCT CCCT***CCGC***CC AC***CCGGAG***GC GGCTGCCGCT AGCCGAGGCC

Sp1 AP2

1081 GGCGCGAGTG CACTGCGAGG CGGGCCGCAG GGAGGGAG**GC GC**GAA**G**AGGG CGCGAGGGTA

E2F (-22)

TSS (-1)

1141 **GC**AGCCAGAG GGAGCCGCCA GCCCTGCCTG CGGATCCCCG CCGGGCGCAT GGGGCGCTTC

1201 GAGCCGGGAC TGCGTGCGGG CCCCGAGGCC AGTTTCCTGC TGTCGTAAGA AGCCGCGCCA

1261 GGACGCCCGC CGGACCCCGA GCTGCTGGGA GG**ATG** **(Translational Start Site)**
