# Supplementary figures and images for: Transcriptional and Post-Transcriptional Mechanisms for Oncogenic Overexpression of Ether À Go-Go K+ Channel
Source: PLoS One. 2011 May 31;6(5):e20362. doi: 10.1371/journal.pone.0020362 (PMC3105031; doi:10.1371/journal.pone.0020362)

Figure S2

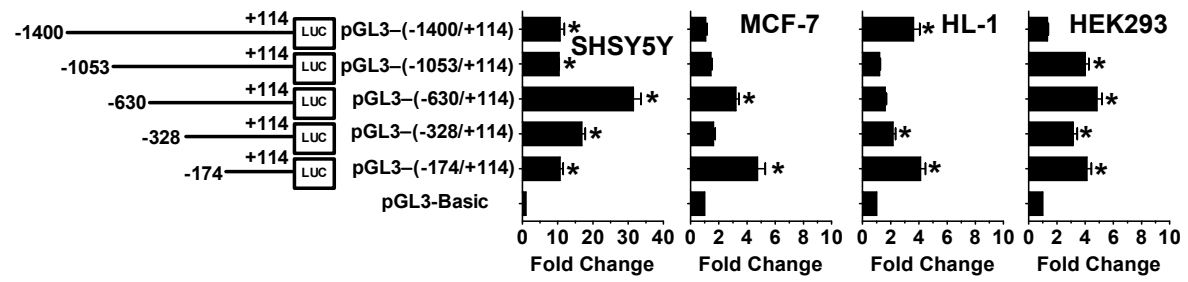

Supplement: Figure S2 — Analysis of the h-eag1 promoter activity in various cell lines. A schematic representation of the 5′ deletion constructs of the h-eag promoter region is shown on the left panel. Nucleotides of fusion plasmids are numbered with respect to the TSS (-1) identified by 5′RACE. Firefly luciferase expression levels were divided by co-expressed Renilla luciferase activity and expressed as relative activity divided by the promoter-less construct (pGL3-Basic). Shown is comparison of the h-eag1 promoter activities expressed in three human cancer cell lines neuroblastoma SHSY5Y, breast cancer MCF-7 and embryonic kidney cell HEK293, and mouse atrial tumor cell line HL-1. The data were averaged from 5 experiments in duplicate for each cell. (PDF) [file pone.0020362.s002.pdf]

Figure S3

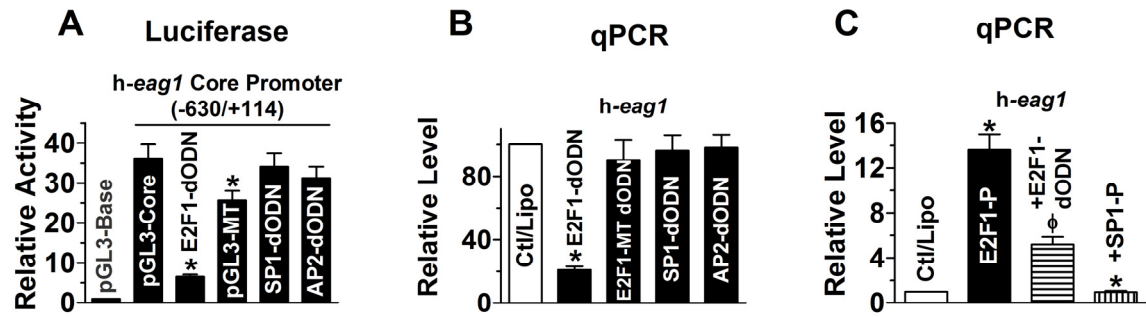

Supplement: Figure S3 — E2F1 as a transactivator of h-eag1 in MCF-7 human breast cancer cell line. (A) Role of E2F1 in driving the h-eag1 core promoter activity. pGL3-Base: h-eag1 promoter-free pGL3 vector for control; pGL3-Core: pGL3 vector carrying the h-eag1 core promoter (a fragment spanning -630/+114); E2F1-dODN, SP1-dODN, and AP2-dODN: the decoy oligodeoxynucleotides targeting E2F1, SP1, and AP2 transcription factors, respectively, co-transfected with pGL3-Core; pGL3-Mutant: pGL3 vector carrying a mutated h-eag1 core promoter. Transfection was carried out using lipofectamine 2000. *p<0.05 vs pGL3-Core; n = 4 for each group. (B) Changes of h-eag1 mRNA level determined by real-time quantitative RT-PCR (qPCR). E2F1-dODN, E2F1-MT dODN, SP1-dODN, or AP2-dODN was transfected alone. Ctl/Lipo: cells mock-treated with lipofectamine 2000; E2F1-MT dODN: the decoy oligodeoxynucleotides targeting E2F1 with mutation at the core region. *p<0.05 vs Ctl/Lipo; n = 4 for each group. (C) Increase in h-eag1 mRNA level by overexpression of E2F1 in MCF-7 cells transfected with the plasmid expressing the E2F1 gene. E2F1-P: pRcCMV-E2F1 expression vector (Invitrogen), the plasmid carrying the E2F1 cDNA. *p<0.05 vs Ctl/Lipo; n = 4 for each group. (PDF) [file pone.0020362.s003.pdf]

Figure S4

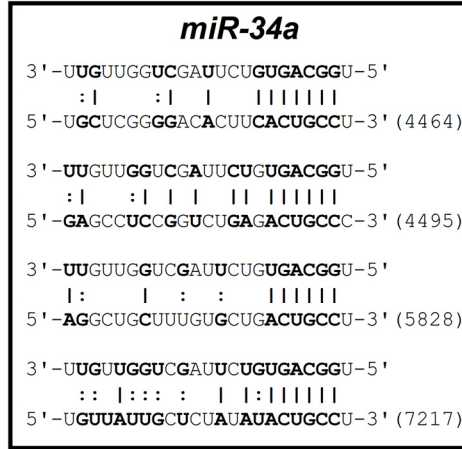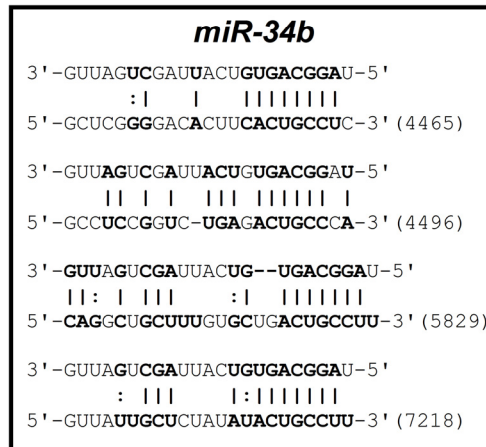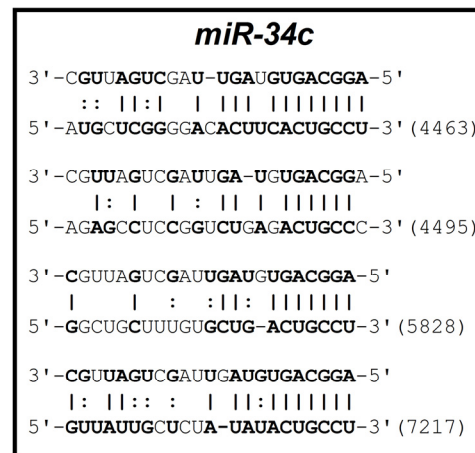

Supplement: Figure S4 — Multiple complementary motifs between each of the three isoforms of has-miR-34 and the 3′UTRs of h-eag1 mRNA (A) and h-erg1 mRNA (B). Matched nucleotides are in boldface and linked by “|”, and wobble matches are indicated by “:”. (PDF) [file pone.0020362.s004.pdf]

Figure S5

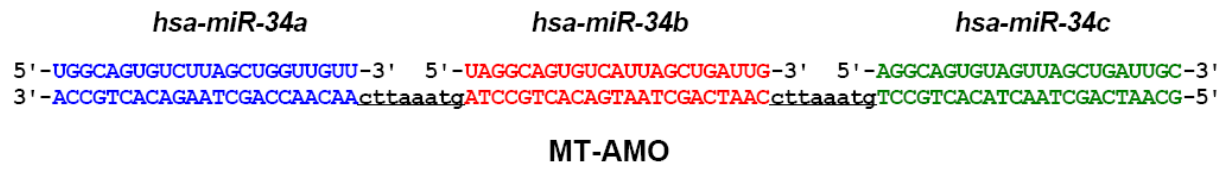

Supplement: Figure S5 — The multiple-target anti-miRNA antisense oligonucleotide fragment (MT-AMO) used to knock down all three different isoforms of has-miR-34 (miR-34a, miR-34b and miR-34c). (PDF) [file pone.0020362.s005.pdf]

Figure S6

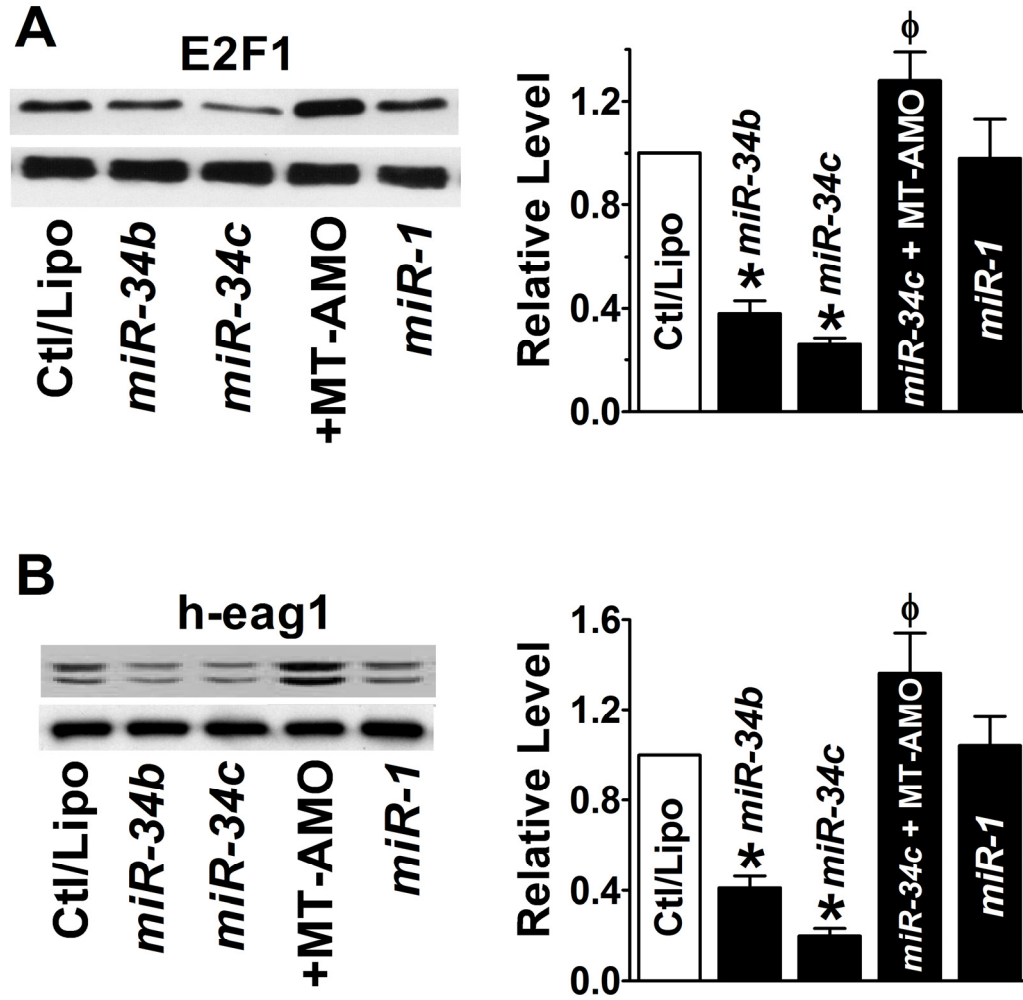

Supplement: Figure S6 — Effects of miR-34b and miR-34c on expression of E2F1 (A) and h-eag1 (B) at the protein level in SHSY5Y cells, assessed by Western blot analysis. Control cells were mock-treated with lipofectamine 2000. miR-1 was used as a negative control. MT-AMO: an antisense oligomer to miR-34a, miR-34b and miR-34c; +MT-AMO: co-app;lication of miR-34c and MT-AMO. *p<0.05 vs Ctl/Lipo; ϕ p<0.05 vs miR-34c alone; n = 4 for each group. (PDF) [file pone.0020362.s006.pdf]

Figure S7

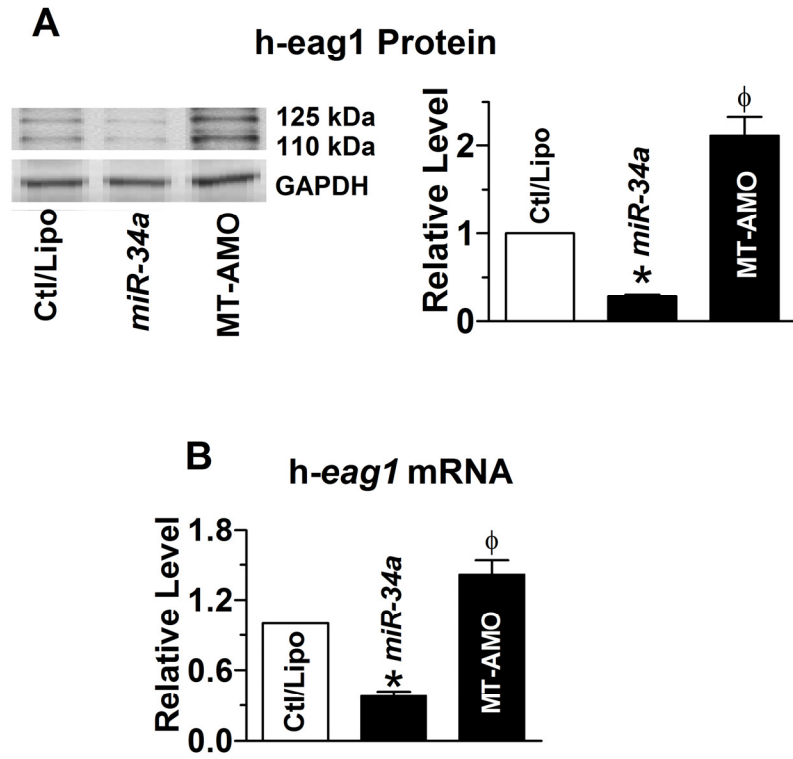

Supplement: Figure S7 — miR-34 as a post-transcriptional repressor of h-eag1 in MCF-7 human breast cancer cells. (A) Western blot analysis revealing repression of h-eag1 protein by miR-34a. *p<0.05 vs Ctl/Lipo; ϕ p<0.05 vs miR-34a alone; n = 5 for each group. (B) Effect of miR-34a on h-eag1 mRNA level. *p<0.05 vs Ctl/Lipo; ϕ p<0.05 vs miR-34a alone; n = 5 for each group. (PDF) [file pone.0020362.s007.pdf]

Figure S8

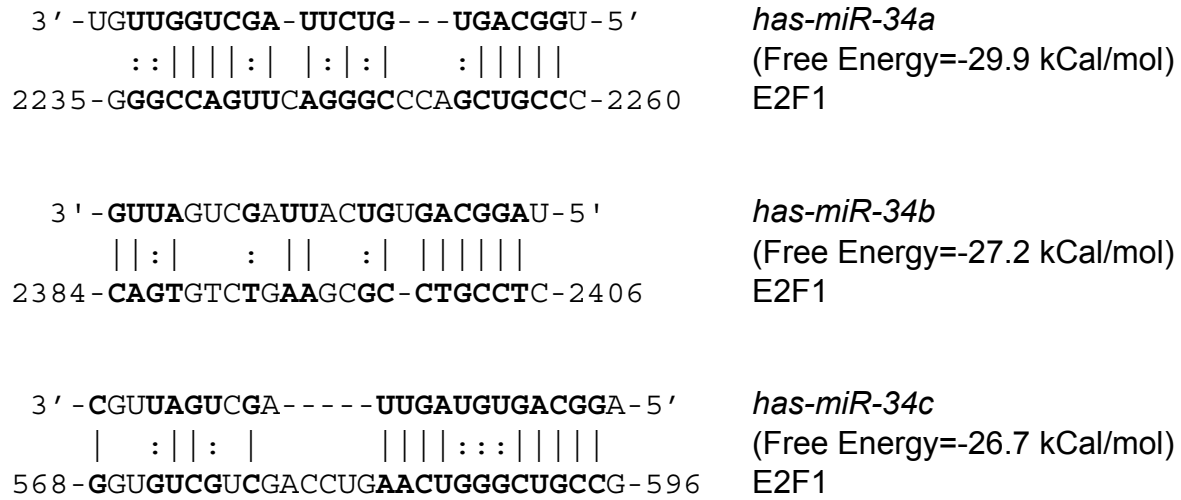

Supplement: Figure S8 — Multiple complementary motifs between each of the three isoforms of has-miR-34 and the 3′UTR of E2F1 mRNA. Matched nucleotides are in boldface and linked by “|”, and wobble matches are indicated by “:”. (PDF) [file pone.0020362.s008.pdf]

Figure S9

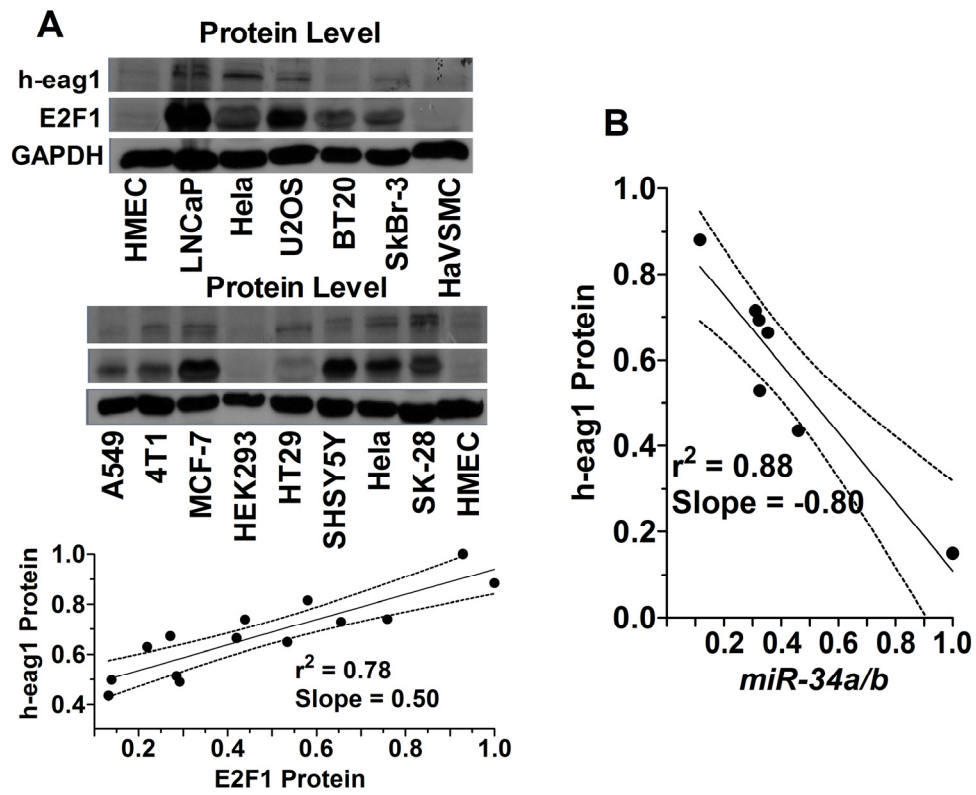

Supplement: Figure S9 — Expression correlations between E2F1 and h-eag1 and between miR-34 and h-eag1. (A) Western blot analysis showing a positive correlation between E2F1 protein and h-eag1 protein levels in various cancer and non-cancer cell lines as specified. Fill circles are experimental data, straight line represents linear regression and dashed lines define the 95% confidence range. The correlation coefficient (r2) and the slope are indicated. (B) qPCR analysis showing an inverse relationship between miR-34a/b and h-eag1 protein levels in HMEB, HaVSMC, SkBr-3, SHSY5Y, U2OS, HT29, and Sk-28 cells. Fill circles are experimental data, straight line represents linear regression and dashed lines define the 95% confidence range. The correlation coefficient (r2) and the slope are indicated. (PDF) [file pone.0020362.s009.pdf]
